# Severe male infertility after failed ICSI treatment-a phenomenological study of men's experiences

**DOI:** 10.1186/1742-4755-8-4

**Published:** 2011-02-04

**Authors:** Marianne Johansson, Anna-Lena Hellström, Marie Berg

**Affiliations:** 1 Department of Obstetrics and Gynaecology, Sahlgrenska University Hospital, Gothenburg, Sweden; 2 Institute of Health and Care Sciences, Sahlgrenska Academy, University of Gothenburg, Gothenburg, Sweden

## Abstract

**Background:**

Male-factor infertility underlies approximately 30% of infertility in couples seeking treatment; of which 10% is due to azoospermia. The development of assisted reproductive technology (ART), enabling the use of epididymal or testicular sperm for fertilization of the partner's oocytes, has made biological fatherhood possible for men with obstructive azoospermia. There is limited knowledge of men's experience of their own infertility. The aim of this study was to describe men's experiences of obstructive azoospermia infertility.

**Methods:**

Eight men with obstructive azoospermia, who had terminated Swedish public health system ART treatment two years previously without subsequent childbirth, were interviewed using a descriptive phenomenological method.

**Results:**

The essence of the phenomenon is expressed with a metaphor: climbing a mountain step by step with the aim of reaching the top, i.e. having a child and thus a family with a child. Four constituents are included (1) inadequacy followed by a feeling of redress (2) marginalisation, (3) chivalry (4) extension of life and starting a family as driving forces.

**Conclusions:**

Knowledge of men's experiences of their own infertility is important as a supporting measure to increase the quality of care of infertile couples. By adopting this facet of gender perspective in fertility treatment guidelines, care can hopefully be optimized.

## Background

Reproduction and childbirth is one of the central themes of life [1], occurring when planned and wished in many but not all cases. The prevalence of infertility is debated. A recent study by Boivin et al. [2]reported that 9% of fertile-age couples suffer either primary or secondary infertility, a prevalence that is confirmed by the authors of this article [3]. Insemination with donor sperm was the only treatment for severe male-factor infertility before the in vitro fertilization (IVF) era [4]. However, while childlessness associated with female-factor infertility has often been solved by IVF, conventional IVF has not yielded the same successful outcome in cases of male-factor infertility [5]. Today, biological fatherhood is possible in certain cases of severe male-factor infertility due to the development of intracytoplasmatic sperm injection (ICSI) [6]. Azoospermia, i.e. the absence of sperm in the ejaculate, is seen in 10% of male-factor infertility cases [7]; it can be either obstructive or non-obstructive/testicular. Surgical sperm retrieval, i.e. spermatozoa collection from either the epididymis or the testis, is required to treat obstructive azoospermia. Epididymal sperm obtained by microsurgical aspiration (MESA) or percutaneous sperm aspiration (PESA) and testicular sperm obtained by surgical excision (TESE) or percutaneous aspiration (TESA) are used in ICSI treatment. Today, reconstruction of the seminal pathways is one of the promising treatments for obstructive azoospermia and parenthood may thus be possible after ICSI or, in some cases, without any further intervention [8]. Surgical treatment has, however, mostly been replaced with ICSI in Sweden. Non-obstructive/testicular azoospermia is caused by impaired or absent spermatogenesis and treatment yields sparse positive results. However, accumulated data have demonstrated that microdissection TESE has brought about outcomes, including conceptions and live births, in couples with non-azoopermia infertility [9,10].

In this paper, we refer to obstructive azoospermia as severe male-factor infertility. This limitation made it possible to study the experiences of men with a specific severe male-factor infertility diagnosis.

Several studies [11,12] have shown that involuntary infertility negatively influences women's quality of life but the results of studies of men's quality of life have not shown the same concordance. Johansson et al. found [3]that couples living without children 4-5.5 years after terminated unsuccessful IVF treatment had a lower quality of life, compared to those living with children, as well as compared to a control group. The childlessness was central in life and subjects reported still suffering grief related to infertility [3]. An Italian study of men's quality of life prior to their first IVF treatment reported no difference, compared to a control group [13] but signs of depression and anxiety were reported in a Brazilian-Canadian study as major predictors of quality of life in men during investigation of infertility [14]. Follow-up studies describe a lower quality of life among men in infertile couples, compared to a control group [15,16] and no differences in quality of life in men and women were found after terminated unsuccessful IVF and the results indicated that involuntary infertility appears to affect quality of life in men more than has been reported previously [16]. Edelmann and Connolly [17] found no evidence of a differential increase in infertility-attributable distress scores for women over time, compared to men. In a meta-analysis concerning gender differences in coping strategies for infertility, Jordan and Reverson [18] found that women used social support- seeking, escape-avoidance, planned problem-solving and positive reappraisal strategies to a higher degree than their male partners. No gender differences were found regarding confrontation, distancing, self-controlling or responsibility-accepting strategies.

In some studies, male-factor infertility is reported not to influence men's psychological well-being and suffering [19,20], but other studies reported increased self-blame and lower self-esteem, compared to men in couples with other infertility diagnoses [21]. Nachtigall et al. reported that men with male factor infertility responded more negatively, emotionally to infertility than men without a male factor; this effect on the gender-specific diagnosis was not seen among women [22]. A sense of profound grief and loss was found among men who had not biologically fathered a child and who had been diagnosed as the sole cause of the couple's infertility problems [23].

There is insufficient knowledge of how men with a severe male-factor infertility diagnosis experience the infertility because there are few studies addressing that issue. The aim of this study was therefore to describe men's experiences of obstructive azoospermia infertility after termination of failed ICSI treatment within the Swedish public health system.

## Methods

### Study population

The subjects were men with a diagnosis of obstructive azoospermia who had terminated ART treatment 2004 and 2005 at the Västra Götaland Regional Reproductive Unit at Sahlgrenska University Hospital in Gothenburg, Sweden. Consultation with a reproductive medicine specialist with andrology competence was undertaken before ART treatment. A maximum of three complete treatments were offered at that time within the Swedish public health system; further treatment must be undertaken in private clinics and be paid for entirely by the couple. Epididymal or testicular sperm were used to fertilize the partner's oocytes. In all cases, ICSI had resulted in fertilized oocytes and embryo transfers (ET) had been performed but none of the treatments had resulted in childbirth. Fourteen men, consecutively identified from the patient database, were informed and invited to participate in the study by mail. Of these, six did not participate: three without explanation, one was ambivalent and finally declined, one was seriously ill and one's partner was pregnant. The eight participants were between 36 and 53 years of age and lived in the western part of Sweden. At the time of the study, two of the participants were living without children, one had children through donor insemination and the others were parents of adopted children. The study was approved by the Regional Ethical Review Board at Gothenburg University, which follows the Helsinki declaration, as revised in 1983.

### Data collection and analysis

A descriptive phenomenological method was used where the concept of lifeworld is central. A person's lifeworld is constituted by the past, present and future. It is important for the researcher to have an open stance, including bracketing one's own pre-understanding in order to discern the meaning of the studied phenomenon [24].

Tape-recorded interviews lasting 60 to 70 minutes were performed in 2006 and 2007, seven in a secluded place in the hospital and one by telephone. The interviewing researcher (MJ) had previously worked with infertility care and treatment but was not doing so at the time of the study. The interviews were relaxed and open and consisted of one simple open request: 'Can you describe your experiences of infertility as extensively as possible?' followed by clarifying questions such as 'What do you mean?' and 'Can you describe that it in greater detail?' The interviews were transcribed into text by the interviewer shortly after they were conducted. The analysis was inspired by Giorgi's phenomenological method [25,26], consisting of four steps. First, the text from all interviews was read and reread in order to obtain an overall sense of the contents. Second, it was reread from a phenomenological reduction perspective and divided into smaller units, whereby changes and transitions in meanings emerged. In the third stage, the meaning units were further analysed until the meanings of the phenomenon were discerned, i.e. meaningful units appeared. In the last step, the meaningful units were transformed into constituents and the essence of the phenomenon materialised. An understanding of the studied phenomenon developed successively during the analysis [25,27].

## Results

The informants had been treated two to thirteen times, either with additional sperm aspiration and ICSI or by donor insemination. One had terminated treatment after two ICSI treatments in the public health system since optimal fertilization was not achieved. The partner of one subject had been pregnant after three of five ART treatments but all pregnancies resulted in miscarriage. The partners of two informants had been treated abroad with donor sperm, resulting in one pregnancy and twin birth. In conclusion: two had no children but were discussing donor sperm insemination; one had children born after donor sperm insemination, the others were parents of adopted children.

### The essence

The essence of the infertility experience in men with severe male-factor infertility can be compared to climbing a mountain with the aim of reaching the top, reaching the different levels step by step: having a child and thus a family with children. The detection of sperm in the epididymis or testicle was the first partial victory, since it entailed the possibility of biological fatherhood, and thus a sense of redress after the previous feeling of inadequacy.

The feeling of outsidership and marginalisation experienced by the informants was related to the focus on the woman in connection with the infertility. The opportunity to process infertility-related emotions was felt limited since the cause of the azoospermia was unknown. This was frustrating; the process toward the "mountaintop" halted and there was a feeling of being caught on a "ledge". Responsibility for loved ones' wellbeing was assumed at the expense of one's own emotions. The men felt worried about their partners and protecting the people close to them. A type of chivalry emerged, based on being strengthened by assuming responsibility, which reinforced the man's sense of self. Different solutions were sought to achieve the goals of children and a family with children, i.e. the "mountaintop".

The phenomenon was described and formed by four constituents, described below.

### Inadequacy, followed by a feeling of redress

A feeling of inadequacy was a prominent part of the experience. Being informed about the absence of sperm in the ejaculate was described as the harshest blow in men's lives and the worst news they had ever received. The possibility of biological fatherhood was perceived as non-existent and feelings of powerlessness and of being different emerged. The masculinity was threatened and it felt like their identity was questioned.

'.nothing that I can do anything about...you can affect other things quite a lot yourself...but I was completely powerless here.' (I 6)

'It's a lot to do with stereotypes of masculine and feminine...whether you want children or not, it's a mark of male quality somehow.' (I 7)

'It's to do with your identity, or rather with your capability, but that's connected with your identity.' (I 8)

In all cases, aspiration of the epididymis or testicle led to the detection of sperm which, together with the information that the sperm appeared to be normal, led to a feeling of redress and to partial return of the lost self-esteem. This information also led to the re-emergence of the hope of biological fatherhood, related to the possibility that ART treatment might work. All was not lost, one level had been reached and a partial victory won. A certain feeling of capability began to materialise.

'They found a reason with me, right, my sperm didn't come out but they were there in my testicle.' (I 4)

'It's important that there actually is sperm there, it's quite important for my self-esteem, it really is.' (I 8)

'It felt more positive at that point...we're on our way now, like, now we're going to make it work.' (I 4)

### Marginalisation

Marginalisation was another central constituent. A sensation of being an outsider emerged; so much was focused on the woman and the man was more of a companion, an unequal partner. The infertility was not perceived as part of the man's world; it was mainly related to the woman. The wish that the woman and the man be more clearly regarded and treated as a couple with a common problem was expressed.

'The IVF process is mainly about the woman, the mother, isn't it....nothing about the man and the man's problems.' (I 5)

'....It feels dodgy to be consulting the Department of Gynaecology....it should be called the Department of Family Medicine' (I 7)

The feeling of marginalisation was aggravated by the fact that the focus of the workup and treatment was not on the man's infertility; the cause of the azoospermia was not investigated. Knowledge about and research concerning azoospermia were perceived as limited, which reduced the possibilities for treatment. Questions about one's own infertility were central and attempts to find explanations were described by the men. Their frustration at not being given an answer to the question of why their ejaculates lacked sperm was substantial. They were left alone with their questions about the infertility. Knowing what the cause was would have made working out infertility-related issues easier.

'...it might go back to my childhood...right around puberty....my friend hit my balls quite hard....so my scrotum went all blue and it swelled up.' (I 5)

'...I'm going to have to live with it (*the uncertainty about the cause of the infertility*)...and I won't rest until I get an answer.' (I 3)

'....so in that regard, I'm still just one big question mark....what's wrong?' (I 1)

### Chivalry

The men cared about their loved ones, giving themselves lower priority and thinking about the woman's treatments and how they might affect her. They had thought about stress and hormonal treatment as well as how a large number of treatments might drain the woman's energy. The situation was perceived as harder for the woman and concern about her emotional reactions to the fertility problems was a central theme. Many aspects of infertility were considered to be more difficult for the woman who could not share her experiences with women who had given birth; pregnancy and delivery were associated with the woman's world. Infertility was more concrete for the woman than the man.

'..three attempts in three years...so it's drained a lot of NN's energy...perhaps not the first time but after the third time.' (I 5)

'I was very worried that NN might be sad.' (I 2)

'The only thing that upsets me is if NN is upset.....she's handled all of this quite well and is very strong now.' (I 3)

'She mourned not being able to experience that, the miscarriage we went through last fall, she gave birth to the fetus, that's how big it was, it was actually a delivery. Afterwards, she thought it had been a fantastic experience that had given her quite a bit, so she was happy that she went through that miscarriage after all.' (I 7)

The psychological pressure was depicted as difficult, but assuming responsibility kept the men going. They described having felt upset, but also having felt that they and their partners had been mutually supportive during the course of the treatments. Supporting other relatives who were disappointed was also portrayed as assuming responsibility. Contact with families with small children was sometimes stressful and informants reported often seeking out families without children.

'I've felt her support when she's gotten over the worst of it. I've never gone into acute crisis since I've been supporting her, your responsibility keeps you going somehow.' (I 4)

'I have to comfort my mum when she gets disappointed or sad or whatever, when nothing happens... and then I have to tell her or somehow soften the blow a bit.' (I 1)

'....well, for one thing I can't cope with too many questions about the problem since it's a very private matter...' (I 3)

'...and we're going through a grieving process the whole time.' (I 1)

The infertility was considered to have strengthened the relationship between the man and the woman in some respects. It had enabled them to talk about family problems, to give each other support during discussions and they had not felt the need to seek counselling from anyone outside the relationship.

'..we've been able to talk about it in the family....so I've never felt the need to talk with any....outsider.....and in some ways you could say it's made our relationship stronger.' (I 3)

### Extension of life and starting a family as driving forces

Starting a family and the perception of belonging to a family were described as central. The possibility of watching a child, created by the man and his partner, grow up, possibly resembling him, was described as an extension of life. Life continuing through one's children, and the importance of leaving something of oneself for posterity, was described as important.

'My wife and I were going to create something together: half from her and half from me.' (I 3)

When the ability to start a family of one's own was threatened, the family of origin-parents and siblings-provided support.

'...it felt quite nice that our parents and siblings help us keep our place as members of a family, otherwise, that's a feeling that might easily have been damaged.' (I 7)

Attempts to solve the family-starting problem were described and the need for knowledge of existing alternatives in order to make decisions was brought up. The analogy of a project proceeding according to plan was made. The advantages and disadvantages related to the different alternatives were elucidated. The difficulty and uncertainty entailed in the choice to start a family by sperm donation or adoption occupied a major part of the men's thoughts. It was not an easy decision. The different possibilities were studied from the child's perspective, but also from the point of view of the man-woman relationship. The decision concerning how to start a family must be well-founded and thoroughly contemplated. The alternative chosen must be a first-hand, rather than a second-hand, choice.

'..you've got to have the whole playing field clear in your mind...otherwise you can't make a decision.' (I 2)

'You struggle with it, don't you?....should you tell the child the truth and should you do it?...would it be better if it was anonymous?....and if you have a child....is it very important to go find your roots?' (I 5)

'We're thinking, 'If we do this now and have a biological baby...what's our little NN going to think about that?' (I 2)

'It's mainly about a lack of balance in...my partner gets.... you know, I don't know how to explain it but....it's about the lack of balance there somehow.' (I 7)

'...no matter which way we chose we wanted it to be a first-hand alternative.' (I 6)

## Discussion

This study may be the first that implements a lifeworld perspective in reporting men's experience of severe male-factor infertility due to obstructive azoospermia. The strength of the study is that it investigates a defined homogeneous group of men with respect to diagnosed male-factor infertility, two years after terminated unsuccessful ART treatment in the public health system. The phenomenological method provides deeper knowledge of human beings' lived experiences [28].

Eight of 14 informed and invited men finally participated. Some of the men who declined participation referred to investigation and treatment as a difficult period that they did not want to mentally relive. The results would probably have been strengthened if all potential informants had participated.

The essence of the phenomenon was described as a process of reaching a mountaintop. Being informed about the azoospermia diagnosis was central in the men's experiences and was described as the toughest information and the hardest blow ever in life. As they perceived it, the possibility of biological fatherhood was non-existent and they themselves could not influence this situation. The feeling of inadequacy was central and the men's sense of masculinity was threatened. A sense of personal inadequacy has also been reported by Webb and Daniluk [23]. Gannon et al. reported that fertility and reproduction were described in the media as closely connected with masculinity and that male-factor infertility was perceived as a particular threat to the conventional views of masculinity [29]. Men with infertility problems felt stigmatized and identified this as relating to their perceived loss of masculinity [22]. According to one study, the most important aspect of infertility was the obligation to fulfil the male role and the social pressure to become a parent [30] and a constituent labelled "procreator" emerged in the men, most strongly in those with suspected male-factor infertility, in a phenomenological study of couples' infertility [31]. The desire to have biological children was described by Langdrige et al. [32] as a need to create something that is part of both the man and the woman and that leads to becoming a family. Biological children may also create a feeling of eternal life [33]. The men in our study experienced a feeling of redress when epididymal or testicular sperm was found, which was important for their self-esteem. The hope of becoming a biological father, aided by ART, returned.

Another constituent in the studied phenomenon was marginalisation. Most investigations and treatments were directed towards the women and the men were assigned a role as companions rather than equal partners. The couples were not regarded as an entity with a common problem. The men had thought about why they had severe male-factor infertility and had sought their own explanations. Not knowing was frustrating and made it more difficult to work out the infertility crisis. Another central part of the men's experiences was their thoughts and concerns about the women's emotional reactions to the infertility and the possible negative physical effects of the treatments. Belgian women with high signs of depression, active coping, avoidance and expression of emotion had lower conception chances [34]. However, this was not found in a study by Anderheim et al [35]. The men saw the infertility as more taxing, emotionally and physically, for the women than for themselves and described a role as a protector and comforter for the women but also for other close relatives, such as the future grandparents. This dimension of responsibility for the partner's wellbeing was also found in an American study among men from 1993 [31], as well as in a Swedish study identifying that the men's reactions were primarily related to the women's reactions rather than considering what childlessness meant to them personally [36]. Another constituent in the phenomenon was the efforts to find solutions to the problem of how to have a family with children, resembling a project with a plan which had to be followed. In order to make constructive decisions the men needed knowledge about different alternative solutions. Decision making, adoption or donor sperm treatment, was not easy and they stated that, regardless of how they chose to have children, it had to be experienced as a first hand choice. There was a major focus on the child's well being in the decision concerning how to create a family with children.

The adoption of a gender perspective and focusing on knowledge of how men and women experience infertility can be one way of increasing the quality of care of couples undergoing infertility treatment. An understanding of the psychosocial issues and coping strategies in infertility, from the women's, the men's and the couples' perspective, is very important when caring for couples [18,34]. Identification of couples who never give up their attempts to reach "the top of the mountain"-having a child and thus a family with children-can be one way to optimize care, as was recommended in a Belgian study among women [34].

This paper focuses on men's experiences of severe male-factor infertility two years after terminated unsuccessful ICSI treatment in the public health system. The findings can be useful when counselling couples with infertility due to obstructive azoospermia and will hopefully contribute to optimizing care. More research focusing on men's experiences, both of absolute (non-obstructive azoospermia) and treatable (obstructive azoospermia) male-factor infertility and of sub-fertility due to defective sperm or sexual inadequacy may be required. The respective experiences after unsuccessful and successful treatment of these different conditions may be diverse and are absolutely of interest to study with methodology resembling that in this study, with results hopefully enabling health-care professionals to optimize caring for these couples.

## Conclusions

In this study, men's experience of obstructive azoospermia after failed ICSI treatment can be summarized in a metaphor: climbing a mountain step by step with the aim of reaching the top, i.e. having a child and thus a family with a child. Four constituents are included (1) inadequacy followed by a feeling of redress (2) marginalisation, (3) chivalry (4) extension of life and starting a family as driving forces. Men's experience of their own infertility is important knowledge when creating treatment guidelines with a gender perspective.

## Competing interests

The authors declare that they have no competing interests.

## Authors' contributions

MJ, ALH and MB participated in the design. MJ interviewed the men and transcribed the interviews into. MJ, ALH and MB participated in the data analyses. MJ prepared the first draughts of the paper. All authors read and made substantial comments and approved the final manuscript

## Funding

The study was supported by grants from Hjalmar Svensson's Research Foundation and Herbert o Karin Jacobsson's Foundation.

## References

[B1] Uddenberg N Uddenberg N Att vilja ha barn- " biologisk drift " eller " socialt tryck" Att få barn En helhetssyn på kris och anpassning när man blir förälder 1976 Lund: Natur och Kultur 15 29

[B2] Boivin J Bunting L Collins JA Nygren KG International estimates of infertility prevalence and treatment-seeking: potential need and demand for infertility medical care Hum Reprod 2007 22 1506 1512 10.1093/humrep/dem046 17376819

[B3] Johansson M Adolfsson A Berg M Francis J Hogström L Janson PO Sogn J Hellström AL Quality of life for couples 4-5.5 years after unsuccessful IVF treatment Acta Obstet Gynecol Scand 2009 88 291 300 10.1080/00016340802705956 19172440

[B4] Schlegel PN Girardi SK Clinical review 87: In vitro fertilization for male factor infertility J Clin Endocrinol Metab 1997 82 709 716 10.1210/jc.82.3.709 9062469

[B5] Tournaye H Devroey P Camus M Staessen C Bollen N Smitz J Van Steirteghem AC Comparison of in-vitro fertilization in male and tubal infertility: a 3 year survey Hum Reprod 1992 7 218 222 1577934 10.1093/oxfordjournals.humrep.a137620

[B6] Palermo G Joris H Devroey P Van Steirteghem AC Pregnancies after intracytoplasmic injection of single spermatozoon into an oocyte Lancet 1992 340 17 18 10.1016/0140-6736(92)92425-F 1351601

[B7] De Croo I Van der Elst J Everaert K De Sutter P Dhont M Fertilization, pregnancy and embryo implantation rates after ICSI in cases of obstructive and non-obstructive azoospermia Hum Reprod 2000 15 1383 1388 10.1093/humrep/15.6.1383 10831574

[B8] Tanrikut C Goldstein M Obstructive azoospermia: a microsurgical success story Semin Reprod Med 2009 27 159 164 10.1055/s-0029-1202304 19247917

[B9] Schlegel PN Testicular sperm extraction: microdissection improves sperm yield with minimal tissue excision Hum Reprod 1999 14 131 135 10.1093/humrep/14.1.131 10374109

[B10] Schiff JD Palermo GD Veeck LL Goldstein M Rosenwaks Z Schlegel PN Success of testicular sperm extraction [corrected] and intracytoplasmic sperm injection in men with Klinefelter syndrome J Clin Endocrinol Metab 2005 90 6263 6267 10.1210/jc.2004-2322 16131585

[B11] Souter VL Hopton JL Penney GC Templeton AA Survey of psychological health in women with infertility J Psychosom Obstet Gynaecol 2002 23 41 49 10.3109/01674820209093414 12061036

[B12] Verhaak CM Smeenk JM Evers AW Kremer JA Kraaimaat FW Braat DD Women's emotional adjustment to IVF: a systematic review of 25 years of research Hum Reprod Update 2007 13 27 36 10.1093/humupd/dml040 16940360

[B13] Ragni G Mosconi P Baldini MP Somigliana E Vegetti W Caliari I Nicolosi AE Health-related quality of life and need for IVF in 1000 Italian infertile couples Hum Reprod 2005 20 1286 1291 10.1093/humrep/deh788 15695309

[B14] Chachamovich JL Chachamovich E Ezer H Cordova FP Fleck MM Knauth DR Passos EP Psychological distress as predictor of quality of life in men experiencing infertility: a cross-sectional survey Reprod Health 2010 7 3 20459694 10.1186/1742-4755-7-3 PMC2878292

[B15] Shindel AW Nelson CJ Naughton CK Ohebshalom M Mulhall JP Sexual function and quality of life in the male partner of infertile couples: prevalence and correlates of dysfunction J Urol 2008 179 1056 1059 10.1016/j.juro.2007.10.069 18206931

[B16] Johansson M Adolfsson A Berg M Francis J Hogström L Janson PO Sogn J Hellström AL Gender perspective on quality of life, comparisons between groups 4-5.5 years after unsuccessful or successful IVF treatment Acta Obstet Gynecol Scand 2010 89 683 691 10.3109/00016341003657892 20302532

[B17] Edelmann RJ Connolly K Gender differences in response to inferility and infertility investigations: real or illusory BJ Health Psychol 2000 5 365 375 10.1348/135910700168982

[B18] Jordan C Revenson T Gender differences in coping with infertility: a meta-analysis J Behav Med 1999 22 341 358 10.1023/A:1018774019232 10495967

[B19] Holter H Anderheim L Bergh C Moller A The psychological influence of gender infertility diagnoses among men about to start IVF or ICSI treatment using their own sperm Hum Reprod 2007 22 2559 2565 10.1093/humrep/dem189 17596276

[B20] Peronace LA Boivin J Schmidt L Patterns of suffering and social interactions in infertile men: 12 months after unsuccessful treatment J Psychosom Obstet Gynaecol 2007 28 105 114 10.1080/01674820701410049 17538818

[B21] Kedem P Mikulincer M Nathanson YE Bartoov B Psychological aspects of male infertility Br J Med Psychol 1990 63 Pt 1 73 80 2331455 10.1111/j.2044-8341.1990.tb02858.x

[B22] Nachtigall RD Becker G Wozny M The effects of gender-specific diagnosis on men's and women's response to infertility Fertil Steril 1992 57 113 121 1730303

[B23] Webb R Daniluk JC The End of the Line: Infertile Men's Experiences of Being Unable to Produce a Child Men and Masulinities 1999 2 6 25 10.1177/1097184X99002001002

[B24] Dahlberg K Dahlberg H Nyström M Reflective lifeworld research 2008 2 Lund: Studentlitteratur

[B25] Giorgi A The theory, practice, and evaluation of the phenomenological method as a qualitative research procedure J Phenomenol Psychol 1997 28 235 260 10.1163/156916297X00103

[B26] Giorgi A Giorgi B Camic P, Rhodes J, Yardley L The Descriptive Phenomenological Psychological Method Qualitative Research in Psychology, Exapanding Perspectives in Methodology and Design 2003 Washington DC: American Psychological Association 243 273

[B27] Giorgi A Concerning the application of phenomenology to caring research Scand J Caring Sci 2000 14 11 15 12035256 10.1111/j.1471-6712.2000.tb00555.x

[B28] Cluett E Bluff R Principles and Practice of Research in Midwifery 2000 Edinburgh, London, New-York, Philadelphia, St Louise, Sydney, Toronto: Baillére Tindal

[B29] Gannon K Glover L Abel P Masculinity, infertility, stigma and media reports Soc Sci Med 2004 59 1169 1175 10.1016/j.socscimed.2004.01.015 15210089

[B30] Hjelmstedt A Andersson L Skoog-Svanberg A Bergh T Boivin J Collins A Gender differences in psychological reactions to infertility among couples seeking IVF- and ICSI-treatment Acta Obstet Gynecol Scand 1999 78 42 48 10.1080/j.1600-0412.1999.780110.x 9926891

[B31] Phipps SA A phenomenological study of couples' infertility: gender influence Holist Nurs Pract 1993 7 44 56 8429069 10.1097/00004650-199301000-00007

[B32] Langdridge D Connolly K Sheeran P A network analytic study of the reasons for wanting a child Journal of Reproductive and Infant Psychology 2000 18 4 321 338 10.1080/713683044

[B33] Gegerfelt Pv Gottlieb C Wilhelmsson J Schoultz Bv Bergqvist A Öppenvårdsgynekologi [Ambulatory care in Gynecology] 2004 2 Stockholm: Liber

[B34] Demyttenaere K Nijs P Evers-Kiebooms G Koninckx PR Coping and the ineffectiveness of coping influence the outcome of in vitro fertilization through stress responses Psychoneuroendocrinology 1992 17 655 665 10.1016/0306-4530(92)90024-2 1287684

[B35] Anderheim L Holter H Bergh C Möller A Does psychological stress affect the outcome of in vitro fertilization? Hum Reprod 2005 20 2969 2975 10.1093/humrep/dei219 16123098

[B36] Wirtberg I His and her childlessness [dissertation] 1992 Stockholm: Karolinska Institutet

